# Direct Detection of Alternative Open Reading Frames Translation Products in Human Significantly Expands the Proteome

**DOI:** 10.1371/journal.pone.0070698

**Published:** 2013-08-12

**Authors:** Benoît Vanderperre, Jean-François Lucier, Cyntia Bissonnette, Julie Motard, Guillaume Tremblay, Solène Vanderperre, Maxence Wisztorski, Michel Salzet, François-Michel Boisvert, Xavier Roucou

**Affiliations:** 1 Département de biochimie, Faculté de Médecine et des Sciences de la Santé, Université de Sherbrooke, Québec, Canada; 2 Département de microbiologie, Faculté de Médecine et des Sciences de la Santé, Université de Sherbrooke, Québec, Canada; 3 PRISM, Laboratoire de Protéomique, Réponse Inflammatoire, Spectrométrie de Masse, EA 4550, SN3, Université Lille 1, Villeneuve d'Ascq, France; 4 Département d'anatomie et de biologie cellulaire, Faculté de Médecine et des Sciences de la Santé, Université de Sherbrooke, Québec, Canada; University of British Columbia, Canada

## Abstract

A fully mature mRNA is usually associated to a reference open reading frame encoding a single protein. Yet, mature mRNAs contain unconventional alternative open reading frames (AltORFs) located in untranslated regions (UTRs) or overlapping the reference ORFs (RefORFs) in non-canonical +2 and +3 reading frames. Although recent ribosome profiling and footprinting approaches have suggested the significant use of unconventional translation initiation sites in mammals, direct evidence of large-scale alternative protein expression at the proteome level is still lacking. To determine the contribution of alternative proteins to the human proteome, we generated a database of predicted human AltORFs revealing a new proteome mainly composed of small proteins with a median length of 57 amino acids, compared to 344 amino acids for the reference proteome. We experimentally detected a total of 1,259 alternative proteins by mass spectrometry analyses of human cell lines, tissues and fluids. In plasma and serum, alternative proteins represent up to 55% of the proteome and may be a potential unsuspected new source for biomarkers. We observed constitutive co-expression of RefORFs and AltORFs from endogenous genes and from transfected cDNAs, including tumor suppressor p53, and provide evidence that out-of-frame clones representing AltORFs are mistakenly rejected as false positive in cDNAs screening assays. Functional importance of alternative proteins is strongly supported by significant evolutionary conservation in vertebrates, invertebrates, and yeast. Our results imply that coding of multiple proteins in a single gene by the use of AltORFs may be a common feature in eukaryotes, and confirm that translation of unconventional ORFs generates an as yet unexplored proteome.

## Introduction

**Figure 1:**
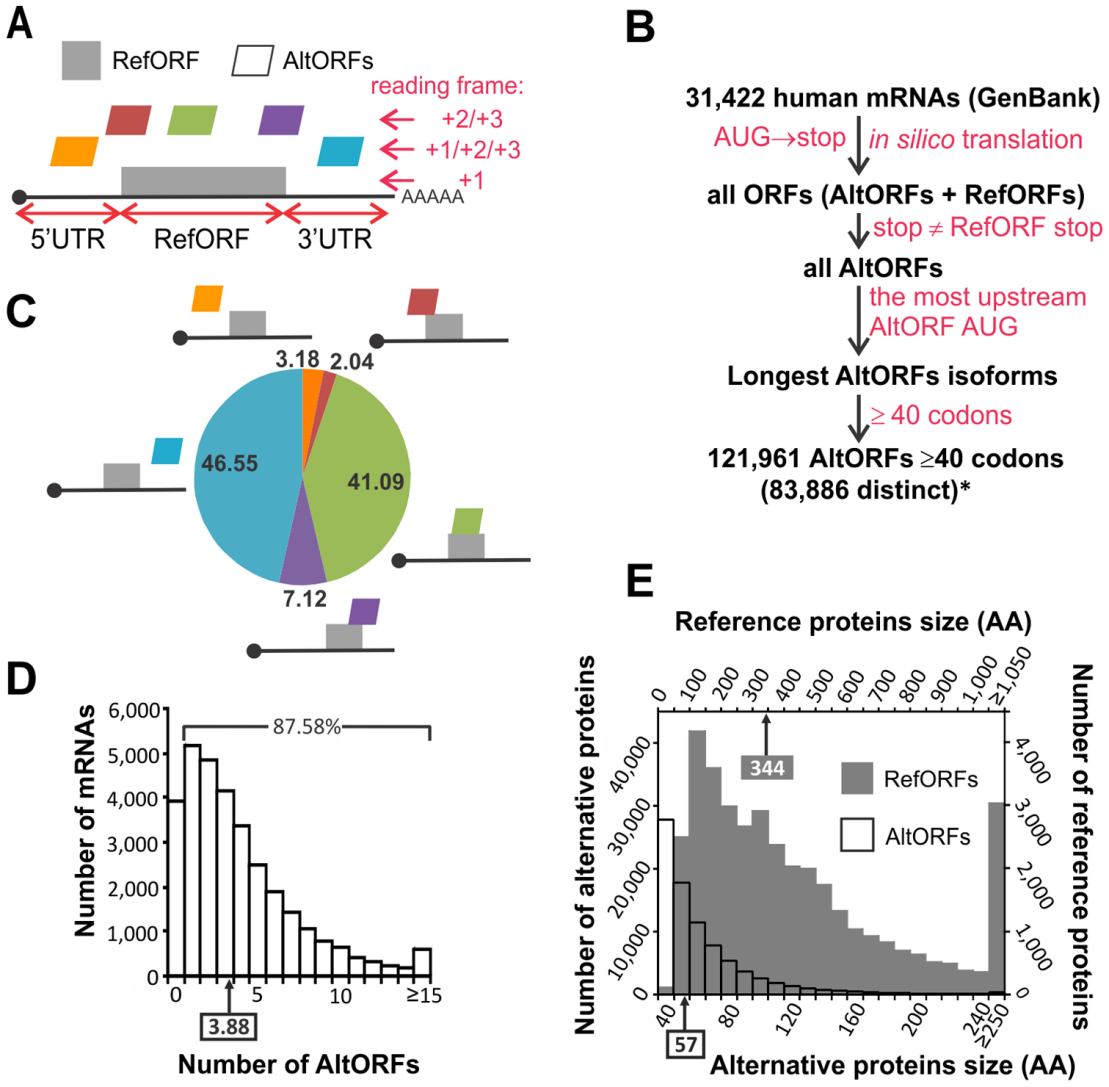
A database to predict AltORFs in human mRNAs. (*A*) A canonical mRNA and its possible AltORFs. The RefORF is the main protein coding ORF annotated in current nucleotide databases. An AltORF is a nucleotide region comprised between an AUG codon and a stop codon distinct from the RefORF and is predicted to encode an alternative protein. AltORFs may be localized in 5′ UTRs, overlapping the 5′UTR and the RefORF, overlapping the RefORF, overlapping the RefORF and the 3′UTR, or in the 3′UTR. (*B*) Representation of the database generation process. Distinct AltORFs number indicates the total number of predicted AltORFs that encode alternative proteins with unique amino acid sequences. Since an AltORF may be present in several transcripts, the total number of AltORFs in the transcriptome exceeds the number of distinct AltORFs. (*C*) Distribution in % of AltORFs. (*D,E*) Distribution of the number of predicted AltORFs per mRNA in (*D*) and the size distribution of AltORFs (empty bars, left and bottom scale) compared to RefORFs (grey bars, right and top scale) in (*E*). Boxes and arrows indicate the median.

The proteome impacts all aspects of health and disease and deciphering the human proteome represents an important challenge in the post-genomic era. A typical fully processed mRNA includes one RefORF and is associated with a reference protein (Fig. 1A). Reference proteins populate current protein databases used to support research in life sciences. For example, protein databases are central to the success of mass spectrometry-based protein identification for the discovery of expression and interaction proteomics, and of biomarkers [1].

Two cellular mechanisms have evolved to increase proteomic diversity by encoding more than one protein per gene, increasing the diversity of the transcriptome or producing more than one protein from a single transcript. Transcriptome diversity [2] is achieved by utilization of alternative promoters [3], reiterative transcription [4], or post-transcriptional processing, including alternative splicing [5], alternative polyadenylation [6] and RNA editing [7]. On the other hand, N-terminal extension [8], ribosomal frameshifting [9], [10] and utilization of multiple coding ORFs in one transcript [11], [12] can generate functional or disease-related proteins.

Out-of-frame alternative translation initiation in the same transcript is used to encode proteins of totally different amino acid composition and is mainly observed in viruses and bacteriophages [13]. This mechanism provides such small organisms with increased coding capacity. Until recently, very few examples were documented in human [11], [12], , and it was assumed such mechanisms were anecdotal in eukaryotes considering the flexibility and coding capacity provided by their large genome. Two recent studies described the discovery of several protein products resulting from translation of non-canonical, alternative open reading frames (AltORFs) [19], [20], present in 5′UTRs, overlapping the RefORFs, or in 3′UTRs. These studies demonstrate that the human proteome is more complex than previously appreciated and suggest that there are many more alternative proteins that remain undiscovered.

Paradoxically, several databases predicting AltORFs in eukaryotes are available [21]. Yet, most of these databases did not actually predict the rare examples of AltORFs translation products documented in humans [11], [12], . Most importantly these databases did not include AltORFs present within UTRs. To address this issue, we generated a database of predicted AltORFs present in mature human mRNAs.

Here, we provide evidence that AltORFs located within UTRs or overlapping the RefORF in a different reading frame are translated and significantly contribute to the human proteome. We demonstrate that basic transfection of cDNAs containing a RefORF and an AltORF results in coexpression of the reference and alternative proteins. In addition, we provide evidence that alternative proteins are conserved with a high sequence identity in vertebrates and invertebrates, suggesting an important function for this unexplored proteome.

## Methods

### Ethics protocol (human tissues)

Ovarian, fallopian tube and endometrial formalin fixed, paraffin-embedded tissues were obtained from the CHRU de Lille pathology department (institutional review approval from the Ethical Research Committee CPP Nord Ouest IV 12/10). The ethical committee considered contacting the patients, often many years after surgery, to be unnecessary and waived the need for written informed consent from the participants.

### AltORFs database

Few databases predicting alternative ORFs (AltORFs) in eukaryotes are available. Yet, most of these databases did not actually predict the very rare examples of out-of-frame alternative translation products documented in humans. Most importantly these databases did not include AltORFs present within UTRs. Other criteria included in previous predictions −conservation among species, presence of an optimal Kozak context around the initiator AUG codon, location within the reference coding sequence− were not taken into account in this study to reduce biases in prediction and generate a comprehensive database.

The AltORF database for each species was built as previously described [21] with minor modifications to the pipeline of Perl scripts that populate a MySQL database, to retain AltORFs regardless of the Kozak context or the location on the mRNA. GenBank entries (http://www.ncbi.nlm.nih.gov/genbank/) were used to generate the AltORF databases for human (release 37), chimpanzee (release 102), mouse (release 103), cow (version 1) and frog (version 1), whereas Ensembl entries (http://www.ensembl.org/) were used to generate the databases for fly (version 70.546), nematode (version 70.230) and yeast (version 70.4). The AltORF databases are provided as excel files (Databases S1−S8).

### Antibodies

Primary antibodies used for western blot were monoclonal anti-HA (Covance), monoclonal anti-GFP (SantaCruz), polyclonal anti-NPTII (Millipore), monoclonal anti-BRCA1 (Bethyl). Secondary antibodies used for western blots were horseradish peroxidase (HRP)-conjugated sheep anti-mouse IgG (NA931V), HRP-conjugated donkey anti-rabbit IgG (NA934V) (GE Healthcare). Primary antibody used for immunofluorescence was monoclonal anti-BRCA1 (Novus Biologicals). Secondary antibodies used for immunofluorescence was Alexa Fluor 568-conjugated goat anti-mouse IgG (Invitrogen).

### Clones

These clones were generated to verify translation initiation at predicted AUG codons, to confirm the coexpression of reference and alternative proteins from the same mRNA, and to visualize the expression of alternative proteins using a GFP tag.

#### Predicted AltORFs with initiation and stop codons in 5′UTRs

*SLC35A4* and *ZNF83* (GenBank mRNA entries NM_080670 and NM_001105552, respectively): gBlocks (IDT) gene fragments containing the 5′UTR sequence up to the last coding nucleotide of their respective AltORF were designed and inserted into the *BamHI* restriction site of pEGFP-N1 using the Gibson assembly kit (New England Biolabs) according to the manufacturer's protocol.

#### Predicted AltORF overlapping the 5′UTR and the reference CDS

*IDH3B* (NM_174856). This construct was synthesized as gBlocks gene fragments (IDT). The construct was assembled and inserted into the *BamHI* site of pcDNA^HA-EGFP+3^ using the Gibson assembly kit. pcDNA^HA-EGFP+3^ was designed to express HA-tagged reference proteins and GFP-tagged alternative proteins with the corresponding AltORF in the +3 reading frame.

#### Predicted AltORFs entirely included in the reference CDS

*NIPA1* (NM_001142275), *BDH2* (NM_020139), *SCARB2* (NM_005506), *LGALS3BP* (NM_005567), *CDC42* (NM_144681), *VEGFC* (NM_005429), *BDKRB2* (NM_000623), *TP53* (NM_000546), and *SRSF1* (NM_006924). For *NIPA1*, *BDH2*, *SCARB2*, *LGALS3BP*, *CDC42* and *VEGFC*, constructs were synthesized as gBlocks gene fragments (IDT), then assembled and inserted into the *BamHI* site of pcDNA^HA-EGFP+2^ using the Gibson assembly kit. For *TP53* and *SRSF1*, inserts were PCR amplified from cDNA containing plasmids (Addgene). The resulting PCR fragments were inserted into the *BamHI* site of pcDNA^HA-EGFP+2^. *BDRKB2* was PCR amplified from a cDNA containing plasmid (clone SC119794, OriGene), and the resulting PCR fragments were inserted into the *BamHI* site of pcDNA^HA-EGFP+3^.

Mutation of the predicted alternative initiation ATG codons to AAG was performed with complementary oligonucleotides containing the mutation, using the QuikChange kit (Stratagene).

Each construct contained the endogenous Kozak sequence (positions −3 to +4) around both the RefORF and AltORF putative initiation codons. 5′ UTR regions were present in the constructs only when the corresponding AltORF was at least partially contained within this region.

The AltMRVI1^EGFP^ fusion was obtained by inserting a gBlock gene fragment containing the AltMRVI1 coding sequence (GenBank mRNA entry NM_001100167, nucleotides 5403 to 5688) into the *BamHI* digested pEGFP-N1 plasmid.

Primers and sequence verified gBlocks gene fragments were purchased from IDT. All constructs were sequenced in both orientations.

### Cell culture and transfection

Human epithelial kidney (HEK293), human cervical cancer HeLa and human colon CCL227, CCL228, CCL233, CRL1459 and HCT116 cells were grown in Dulbecco's Modified Eagle's Medium supplemented with 10% Fetal Bovine Serum and penicillin/streptomycin. Human colon cell lines CCL227, CCL228, CCL233, CRL1459, and HCT116 were purchased from ATCC. Transfections were carried out using GeneCellin according to the manufacturer's protocol (BioCellChallenge).

### Protein sample preparation, immunoprecipitation and western blot

For validation of alternative proteins expression by HA- and GFP-tagging, HeLa cells were grown in 12-well plates for 24 h and were then transfected as described above. Cells were rinsed with PBS and lysed in SDS-PAGE sample buffer (0.5% SDS (w/v), 1.25% 2-β-mercaptoethanol (v/v), 4% glycerol (v/v), 0.01% bromophenol blue (w/v), 15 mM Tris-HCl, pH 6.8). After electrophoresis, proteins were transferred to PVDF membranes and detected by western blot using anti-HA (Covance, 1/1000), anti-GFP (Santa Cruz, 1/1000), and anti-NPTII (Millipore, 1/1000) antibodies.

For immunoprecipitation of AltMRVI1^EGFP^, HEK 293 cells were grown in 100 mm plates for 24 h before transfection. After 24 h, cells were rinsed twice with ice-cold PBS and lysed in 1 mL NETN buffer (50 mM Tris-HCL, pH 8.0, 0.15 M NaCl, 1 mM EDTA, 0.5% NP-40, with protease inhibitors (Roche) and protein phosphatase inhibitors (Thermo Scientific)) for 15 min at 4°C. Nuclei were broken by successive passing in 18G, 20G, 21G and 25G needles, and the lysate was centrifuged at 15,000× g for 15 min at 4°C. Protein concentration was quantified using BCA protein assay reagent (Pierce). Ten μL GFP-Trap agarose beads (ChromoTek) were used for GFP immunoprecipitation of 0.75 mg sample (1 mg/mL in NETN buffer) during 1 h at 4°C. The beads were then washed three times with 1 mL of NETN buffer, and the bound proteins eluted by incubating for 5 min at 95°C in SDS-PAGE sample buffer. Proteins were detected by western blot using anti-GFP (Santa Cruz, 1/1000), and anti-BRCA1 (Bethyl, 1/1000) antibodies.

Protein samples preparation and in-gel digestion of proteins from normal colon tissue and colon cell lines prior to LC-MS/MS analysis were performed as previously described [22]. For LC-MS/MS analysis of proteins contained between the 4.6 and 10 kDa markers of an 1D SDS-PAGE, HeLa cells were lysed in a buffer containing 4% SDS/100 mM DTT/100 mM Tris-HCl pH 7.6, and proteins alkylated in 50 mM iodoacetamide. Eight hundred µg of sample was migrated in 8 different lanes (100 µg per lane) of a 4−12% Bis-Tris polyacrylamide NuPAGE Novex gel (Invitrogen), and stained with SimplyBlue Safestain (Invitrogen). In each lane, a single gel slice (between the 4.6 and 10 kDa markers) was cut and processed for in-gel digestion by trypsin [23]. Tryptic peptides were extracted by 1% formic acid followed by 100% acetonitrile before lyophilization in a SpeedVac and resuspension in 1% formic acid prior to LC-MS/MS.

### Immunofluorescence and confocal microscopy

Immunofluorescence and confocal microscopy analyses were carried out as previously described [24], [25].

### LC-MS/MS analyses of HeLa cell lysates, colon cell lines and colon tissues

LC-MS/MS analyses of HeLa cell lysates, colon cell lines (CCL227, CCL228, CCL233, CRL1459 and HCT116 cells) and colon tissues were performed as previously described [22].

### LC-MS/MS analyses of cancerous ovarian, normal ovarian, cancerous fallopian tube and normal endometrial tissues

Formalin fixed, paraffin-embedded tissues were obtained from the CHRU de Lille pathology department (institutional review approval CPP Nord Ouest IV 12/10). For the histological imaging prior to LC-MS/MS analysis, 4 µm-thick tissue sections were cut from the formalin-fixed, paraffin-embedded (FFPE) whole-mount ovarian tissue blocks. The sections were placed on ITO-coated slides and heated for 60 min at 58°C [26]. The tissue was counterstained with hematoxylin, eosin and safran (HES), dehydrated using graded ethanol solutions, and air-dried for histological examination by our staff pathologist. The tissues appeared heterogeneous and contained cancerous, hyperplastic, and normal regions, with stromal tissue in each region [26]. The International Federation of Gynecology and Obstetrics (FIGO) stages were determined.

For tissue de-waxing, sections (10 µm) were generated using a microtome and were applied to conductive glass slides that were coated with ITO (indium tin oxide) on one side. The paraffin was removed by submersion in toluene twice for 5 min, followed by a light rehydration in ethanol baths (100%, 96%; 70% and 30%) before the slides were dried in a desiccator at room temperature [26], [27].

Citric acid antigen retrieval was performed by immersing the slides in 10 mM of citric acid for 20 min at 90°C and then drying them in a desiccator for 10 min. Prior to the enzymatic digestion, the slides were incubated in 10 mM NH_4_HCO_3_ twice to remove the remaining antigen retrieval solution and to condition the tissue for effective enzyme activity.

Ten milliliters of a solution of 40 mM trypsin in 50 mM ammonium bicarbonate was dropped onto each tissue region of interest. The slides were then incubated for 4 hours at 37°C in a customized humidity chamber (a 10 cm×15 cm box filled with water to one quarter of the box height and placed in a 37°C incubator). After trypsin digestion, 10 µL of a 10 mg/mL HCCA solution in aqueous TFA 0.1%/ACN (3:7) was dropped onto each section [28], [29].

Trypsin-digested peptides were manually extracted from specific tissue regions. Using a micropipette, specific regions were subjected to 20 successive washes with 100 μL of a solution of 80% ACN in water. The extract solution was then submitted to freeze-dried with a SpeedVac (Savent). The dried peptides were then re-dissolved in 10 µL of 0.1% TFA. Salts were removed from the solutions, and peptides were concentrated using a solid-phase extraction procedure with the Millipore ZipTip device with a final 10 μL 80% ACN elution solution. The solution was then dried again using the SpeedVac. Dried samples were solubilized in water/5% acetonitrile/0.1% formic acid. Samples were separated by online reversed-phase chromatography using a Thermo Scientific Proxeon Easy-nLC system equipped with a Proxeon trap column (100 μm ID ×2 cm, Thermo Scientific) and a C18 packed-tip column (100 μm ID ×15 cm, NikkyoTechnos Co. Ltd). Peptides were separated using a 5%−40% gradient of acetonitrile over 110 minutes at a flow rate of 300 nL/min. The LC eluent was electrosprayed directly from the analytical column and a voltage of 1.7 kV was applied via the liquid junction of the nanospray source. The chromatography system was coupled to a Thermo Scientific Orbitrap Elite mass spectrometer. The mass spectrometer was programmed to acquire in a data-dependent mode. The survey scans were acquired in the Orbitrap mass analyzer operated at 120,000 (FWHM) resolving power. A mass range of 400 to 2000 m/z and a target of 1E6 ions were used for the survey scans. Precursors observed with an intensity over 500 counts were selected “on the fly” for ion trap collision-induced dissociation (CID) fragmentation with an isolation window of 2 amu and a normalized collision energy of 35%. A target of 5000 ions and a maximum injection time of 200 ms were used for CID MS^2^ spectra. The method was set to analyze the 20 most intense ions from the survey scan and a dynamic exclusion was enabled for 20 s.

### LC-MS/MS analyses of lung tissue, cerebrospinal fluid, urine, plasma and serum

Raw data were obtained from the PeptideAtlas online repository [30] under the following accession numbers: lung (PAe001771), cerebrospinal fluid (PAe001777), urine (PAe000761 and PAe000763), plasma (PAe000846), serum (A: PAe000135 ; B: PAe000347 ; C: PAe000281 ; D: PAe000331, PAe000332, PAe000333, PAe000334, PAe000335, PAe000337, PAe000338).

### Bioinformatics analysis

Quantitation was performed using the program MaxQuant version 1.2.2.5 [31], [32]. The derived peak list generated by Quant.exe (the first part of MaxQuant) was searched using Andromeda as the database search engine for peptide identifications against the human GenBank protein entries (release 37) containing 37,390 proteins, to which the 83,886 predicted alternative proteins and 175 commonly observed contaminants and all the reversed sequences had been added. The first search mass tolerance was set to 20 p.p.m. and main search mass tolerance was 6 p.p.m. Enzyme was set to trypsin/p with 2 missed cleavages. Carbamidomethylation of cysteine was searched as a fixed modification, whereas N-acetyl protein and oxidation of methionine were searched as variable modifications. For the serum sample PAe000347, asparagine deamidation was added as a fixed modification, and glutamine deamidation as a variable modification. For analysis of fractionated HeLa cells, colon cell lines, colon tissue, <10 kDa HeLa cells proteins, and paraffin-embedded human tissues, identification was set to a false discovery rate of 5%, determined by the use of a reverse database (similar results were obtained by using a randomly generated database). To achieve reliable identifications, only the proteins associated with a PEP value of less than 0.05, and identified with at least one unique peptide were retained. Indeed, the detection of more than one tryptic fragment for small proteins is unlikely [19], though we detected at least two tryptic fragments for 148 alternative proteins (Table S4). For analysis of lung tissue and fluids, identification was set to a false discovery rate of 1%, with at least one unique peptide. Protein isoforms and proteins that cannot be distinguished based on the peptides identified are grouped and displayed on a single line, but with a single GenBank accession number (Tables S1−S3).

Accession numbers for detected alternative proteins are provided in Table S4 and are publicly available on the nucleotide database of the European Bioinformatics Institute website (http://www.ebi.ac.uk/).

The conservation analyses of alternative and reference proteins were carried out with BLASTP [33] (version 2.2.27+). The AltORFs databases and reference databases from different species were searched against the corresponding human database with an expectation (E) value cutoff of ≤ 10^−4^. Only the best matching hit for each predicted protein was considered. The reference protein databases match the GenBank and Ensembl releases used to generate the AltORFs databases.

## Results

To generate our database, we defined AltORFs as ORFs located in a non-canonical reading frame of the RefORF, in the 5′ and 3′UTR regions of an mRNA, or partially overlapping with both the RefORF and an UTR region (Fig. 1A). We based our prediction algorithm on characteristics of known AltORFs (AUG as TIS, alternative stop codon different from the RefORF stop codon), and added a size cut-off of 40 codons to keep the database to a reasonable list of polypeptides readily analysable by LC-MS/MS or detectable by SDS-PAGE (Database S1; Fig. 1B). Criteria included in previous predictions −conservation among species, presence of an optimal Kozak context around the initiator AUG codon, location within the reference coding sequence− were not taken into account in this study because experimental evidence indicate that these criteria are not necessarily required for an AltORF to be expressed [11], [12]. Our database predicts 83,886 unique AltORFs with a minimum size of 40 codons (Fig. 1B). Most predicted AltORFs overlap RefORFs (41.09%) or populate 3′UTRs (46.55%) (Fig. 1C). The majority of mRNAs (87.58%) have at least one predicted AltORF (Fig. 1D), and there is an average of 3.88 AltORFs for each mRNA. These proportions are in agreement with the number of detectable translation initiation sites (TIS) determined by ribosome profiling [34], [35]. Most predicted AltORFs have less than 100 codons, and the median alternative protein length is 57 amino acids, compared to 344 for the conventional proteome (Fig. 1E).

**Figure 2:**
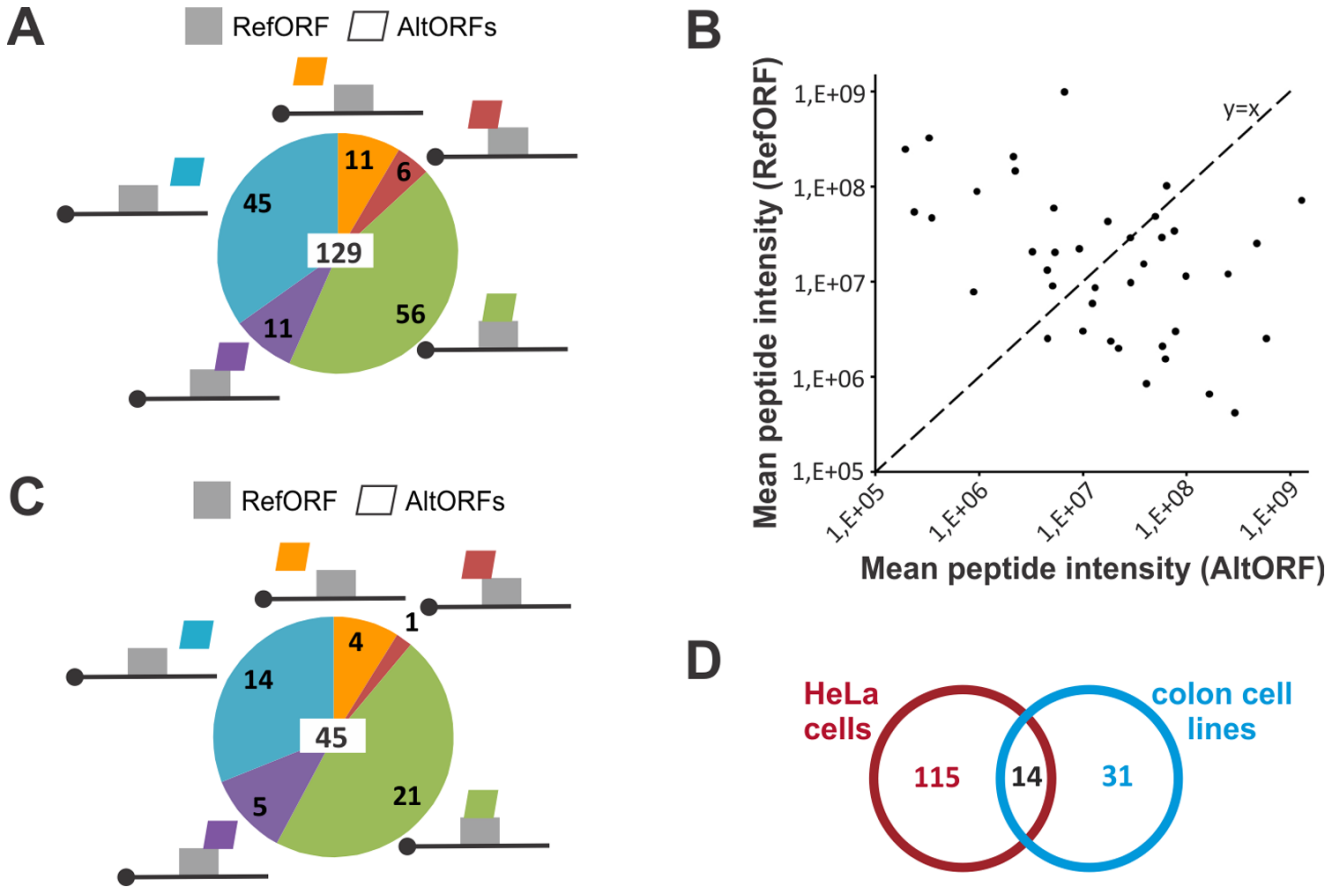
Endogenous expression of alternative proteins in cultured cells. (*A*) Alternative proteins expression was analyzed by LC-MS/MS in HeLa cells, and a schematic distribution of AltORFs in absolute numbers is shown. There were a total of 129 identified alternative proteins (indicated in the center). (*B*) Average peptide intensity plot of both the reference and alternative proteins that were co-expressed from 42 genes. (*C*) Same as (*A*) with colon cell lines. (*D*) Venn diagram showing the number of alternative proteins identified in HeLa cells and colon cell lines. The overlap identifies a common list of 14 alternative proteins.

Using this novel alternative protein database as well as GenBank protein entries, we analyzed a HeLa cells proteomic data set we had previously generated by LC-MS/MS [22]. A total of 68,035 peptides from 5,558 reference proteins and 280 peptides from 129 alternative proteins were identified (Table 1; Table S1; Fig. 2A). The mean sequence coverage for reference and alternative proteins was 28.8% and 32.3%, respectively. Overall, alternative proteins represented 2.27% of the total identified proteins. This result clearly shows that the contribution of alternative proteins to the proteome, and thus the number of multiple coding genes, has been overlooked. It is noteworthy that alternative proteins coding sequences are spread across the different regions of mRNAs in agreement with the predicted distribution (compare Fig. 2A and 1C). Co-expression of an alternative protein and its reference protein was observed for 42 genes (Table S1). For each of these genes, the average peptide intensity plot of both the reference and alternative proteins revealed large variations in co-expression ratio (Fig. 2B), indicating that a reference protein might not always be the main protein product of a gene. To confirm the expression of alternative proteins in cell lines different from HeLa cells, we performed LC-MS/MS on human colon cell lines and identified 45 alternative proteins (Table 1; Table S1; Fig. 2C). AltORFs associated with these 45 proteins were distributed within UTRs and RefORFs with frequencies comparable to those observed in HeLa cells (compare Fig. 2C and 1C). Comparative analysis of alternative proteins detected in both HeLa cells and colon cell lines indicated that 14 are expressed in at least two cell lineages (Fig. 2D). This is more than expected by chance (Fisher's exact test, p  =  3.196e-29).

**Table 1:** Summary of LC-MS/MS analyses of human samples.

Sample	Alternative proteins (peptides)	Reference proteins (peptides)	Alternative proteins (% of total identified proteins)^a^	% sequence coverage (alternative/reference)
Fractionnated HeLa cells	129 (280)	5,558 (68,035)	2.27	28.8/32.3
Colon cell lines	45 (63)	3,512 (39,285)	1.27	17.1/28.0
Colon tissue	13 (15)	1,985 (16,068)	0.65	19.8/23.7
Normal ovary	4 (4)	1,389 (6,224)	0.29	19.5/12.7
Serous ovary	6 (7)	1,935 (8,372)	0.31	17.7/11.8
Serous fallopian tube	3 (3)	1,379 (5,090)	0.22	8.8/11.0
Endometrioid ovary	8 (8)	1,451 (5,762)	0.55	13.0/11.7
Normal endometrium	3 (3)	1,240 (4,649)	0.24	9.3/10.1
<10kDa HeLa	14 (18)	44 (109)	24.14	25.4/35.3
Lung^b^	40 (60)	2,373 (16,987)	1.66	19.3/18.4
Cerebrospinal fluid^b^	16 (22)	266 (1,963)	5.67	18.4/19.0
Urine^b^	47 (50)	754 (2,898)	5.87	24.9/15.5
Plasma^b^	90 (96)	70 (92)	56.25	34.7/4.9
Serum A^b^	311 (326)	192 (351)	61.83	23.4/3.5
Serum B^b^	269 (293)	339 (847)	44.24	28.4/9.7
Serum C^b^	158 (160)	279 (977)	36.16	43.1/8.0
Serum D^b^	230 (248)	190 (365)	54.76	N/A
Serum total^b^	928 (1,002)	754 (2,066)	55.17	N/A
**TOTAL**	1,259 (1,525)	7,341 (85,311)	14.64	N/A

a Total identified proteins  =  alternative + reference proteins.

b PeptideAtlas accession numbers associated with lung, cerebrospinal fluid, urine, plasma and serum samples are indicated in the Methods section. The Serum total line indicates the sum of distinct proteins detected in Serum A, B, C and D.

SDS-PAGE in combination with LC-MS/MS is generally limited to the analysis of proteins above 10 kDa, and a low molecular weight is a known limitation in protein identification by MS [36], [37]. Since the majority of the predicted alternative proteome is composed of proteins less than 90 amino acids long which have a predicted molecular weight below 10 kDa (Fig. 1E), it is not surprising to have detected much more peptides corresponding to the conventional proteome compared to the alternative proteome. To further assess the abundance of the alternative proteome compared to the conventional proteome, HeLa cells proteins were separated by 1-D SDS-PAGE, and one gel slice between the 4.6 and 10 kDa markers was trypsin digested. The resulting peptides were analyzed by LC-MS/MS. A total of 44 reference and 14 alternative proteins were detected, and alternative proteins represented 24.14% of the total identified proteins (Table 1; Table S1), thus showing that alternative proteins are enriched in the pool of small cellular proteins. The detection of alternative proteins with MW between 4.78 and 9.49 kDa (Table S1) is further proof that peptides were not misassigned and that these alternative proteins are actually expressed.

Next, we tested the expression of alternative proteins in a variety of human tissues by LC-MS/MS. First, we analyzed normal colon and lung tissues and detected 13 and 40 alternative proteins respectively (Table 1; Table S2). In a second set of experiments, we analyzed ovarian cancer tissue areas and normal areas from the same formalin fixed, paraffin-embedded tissue section of two patients, one presenting endometrioid ovarian cancer and the second presenting a serous ovarian cancer (Fig. S1). A total of 19 alternative proteins were identified in the normal endometrium, endometrioid ovary, serous ovary, normal ovary, and serous fallopian tube (Table 1; Table S2). We completed these proteomic studies with human fluids, including cerebrospinal fluid, urine, plasma, and serum, identifying 16, 47, 90, and 928 alternative proteins in each fluid respectively (Table 1; Table S3). Strikingly, alternative proteins represent approximately 55% of the proteins identified in plasma and serum (Table 1). Overall, we detected a total of 1,259 alternative proteins (Table 1), and 47 were expressed in different cell lines and/or tissues (Table S4).

**Figure 3:**
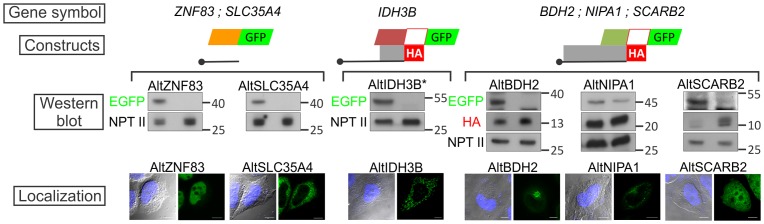
Transfection of tagged constructs validate the expression and translation initiation site prediction of alternative proteins detected by LC-MS/MS. Top diagrams represent the constructs used to detect the co-expression of HA-tagged reference and GFP-tagged alternative proteins by western blot analyses of HeLa cell lysates. GFP is inserted before the alternative stop codon in frame with the AltORF. The black line represents a specific region of the endogenous mRNA. For AltORFs located in 5′UTRs of *ZNF83* and *SLC35A4*, the constructs do not contain the RefORF since the insertion of GFP may prevent the expression of the downstream RefORF. For AltORFs overlapping the 5′UTR and the RefORF (*IDH3B*), and for AltORFs overlapping the RefORF (*BDH2*, *NIPA1*, *SCARB2*), the HA tag was introduced before the GFP tag in frame with the RefORFs. Western blots show the co-expression of reference and corresponding alternative proteins in cell lysates with anti-HA and anti-GFP antibodies, respectively. The left and right lanes are cell lysates from cells expressing a construct with a normal alternative initiation AUG codon or with an inactivated alternative initiation AAG codon, respectively. NPTII, encoded in the expression plasmid, was used as a transfection control. Molecular weight markers in kDa are indicated on the right. Bottom panels show confocal/DIC images with the various cellular distributions of GFP-tagged alternative proteins. Nuclei were stained with Hoechst. Scale bar: 10 μm. * The reference protein was not detected due to the small size (<3 kDa) of the truncated HA-tagged reference IDH3B protein.

In accordance with the scanning model of translation initiation, we used the first AUG rule in order to predict the TIS of AltORFs present in our database. Since other non-AUG codons can be used as TIS [34], we tested the reliability of our TIS prediction for the alternative proteins previously detected by two independent methods. First, the detection of N-acetylated peptides, a modification specific to protein N-termini [38], in 889 out of the 1,259 total alternative proteins detected throughout our different LC-MS/MS experiments allowed us to determine that in most cases (886/889), the alternative TIS predicted in our database was correct (Table S4). Second, we randomly selected and tested the co-expression of 6 alternative proteins and their corresponding reference proteins from the 129 alternative proteins detected by LC-MS/MS in the fractionated HeLa cells lysate. A strategy based on the transfection of constructs with two tags, an HA tag in frame with the reference protein and a GFP tag in frame with the alternative protein, was used to report the co-expression of both proteins in transfected cells (Fig. 3). The corresponding alternative proteins were all detected by both western blot and GFP fluorescence. Importantly, inactivating mutations (AUG to AAG) of the predicted alternative TIS significantly reduced their expression (Fig. 3).

**Figure 4:**
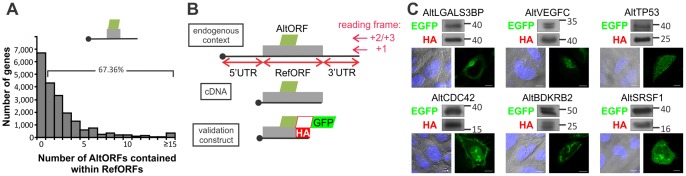
Co-expression of alternative and reference proteins in cDNA transfection experiments is common. (*A*) Distribution of the number of predicted RefORFs-contained AltORFs per gene in the human genome. Top, schematic representation of a mRNA with a RefORF (grey)-containing AltORF (green). By definition, RefORFs are present in the +1 reading frame and AltORFs are present in the non-canonical +2 and +3 reading frames. (*B*) Strategy to detect the co-expression of reference and alternative proteins in cDNA transfection experiments. HA and GFP tags permit the detection of reference and alternative proteins, respectively. Top, graphical representation of a mRNA with a RefORF-contained AltORF. Middle, typical cDNA construct used in transfection experiments. Bottom, representation of constructs used in (*C*). (*C*) Western blot analyses of HA-tagged LGALS3BP (Lectin galactoside-binding soluble 3 binding protein), VEGFC (vascular endothelium growth factor), p53 (cellular tumor antigen p53), CDC42 (cell division cycle 42), BDKRB2 (bradykinin receptor), and SRSF1 (serine/arginine-rich splicing factor 1), and their respective GFP-tagged alternative proteins using anti-HA and anti-GFP antibodies (top panels). Bottom panels show the cellular distribution of alternative proteins by confocal fluorescence microscopy (differential interference contrast and Hoechst, left panels; GFP, right panels). Scale bar: 10 μm.

Transfection of cDNAs in cultured cells is a routine technique in most laboratories. The possible unnoticed co-expression of an alternative protein with the reference protein could be a major issue, as 67.36% of human protein coding genes are predicted to have at least one AltORF contained within the RefORF (Fig. 4A). We selected 6 well studied RefORFs from the AltORFs database, including the tumor suppressor p53 (Fig. 4C). The strategy to detect the co-expression of reference and alternative proteins is shown in Fig. 4B. After transfection, we determined that each cDNA led to the constitutive co-expression of the alternative and reference proteins as observed by western blot and fluorescence (Fig. 4C). Diverse subcellular distributions could be observed among the tested constructs (Fig. 4C, see also Fig. 3), suggesting a variety of possible functions associated with alternative proteins.

**Figure 5:**
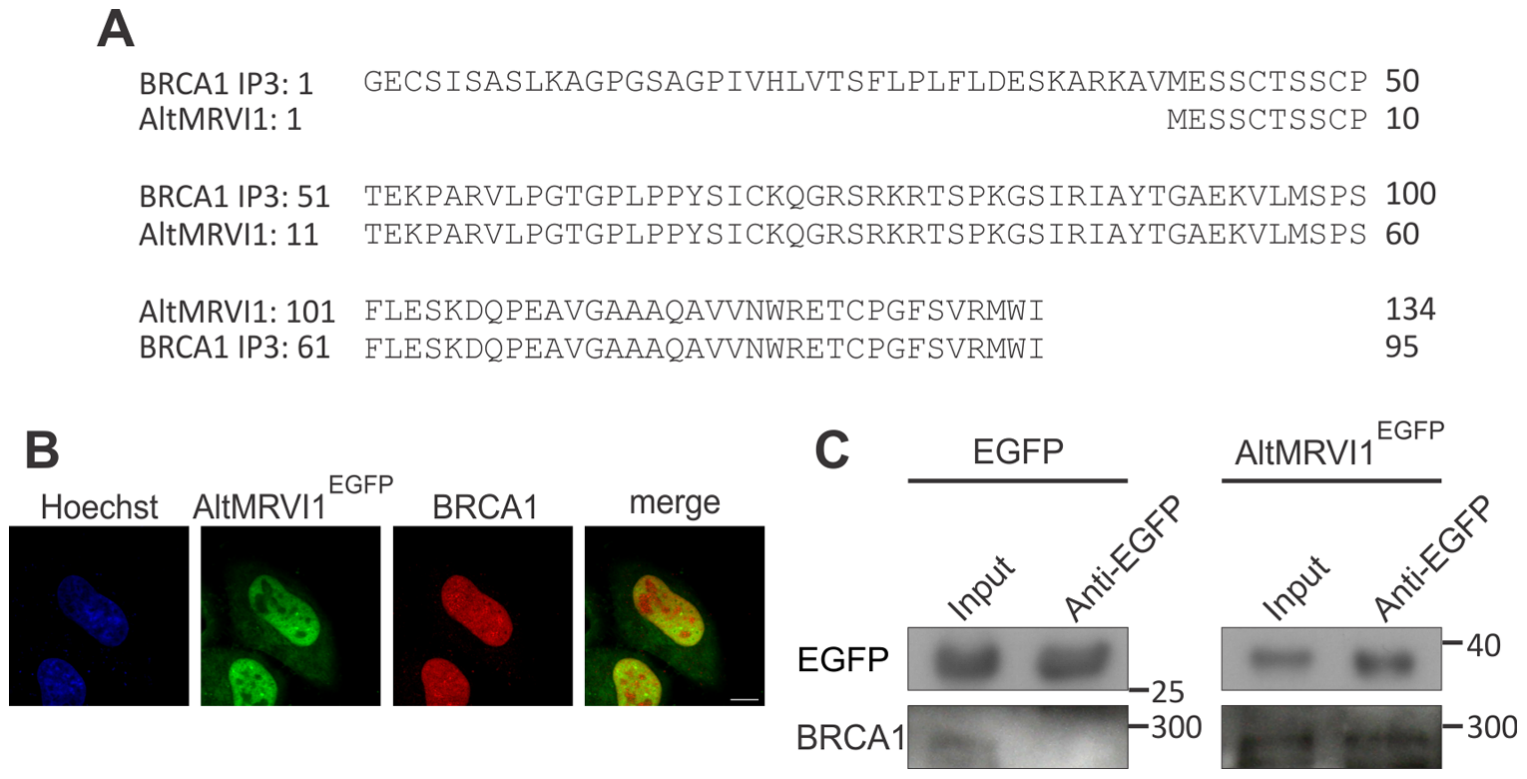
The alternative protein AltMRVI1 interacts with BRCA1. (*A*) Sequence alignment between the third BRCA1 interacting protein 3 (BRCA1 IP3) identified by Liu et al by Y2H [40] and AltMRVI1. (*B*) Localization of BRCA1 and AltMRVI1^EGFP^ in the nucleus. HeLa cells transfected with AltMRVI1^EGFP^ (green channel) were immunostained with anti-BRCA1 (red channel) antibodies. Nuclei were stained with Hoechst (blue channel). Scale bar, 10 μm. (*C*) Co-immunoprecipitation with anti-GFP antibodies was performed using lysates from HeLa cells expressing AltMRVI1^EGFP^. Molecular weight markers in kDa are indicated on the right.

Many cDNA clones identified in large scale screening assays, including yeast two-hybrid (Y2H) studies do not match any known protein of the conventional proteome because they represent out-of-frame clones [39], [40]. In Y2H, these unknown interacting proteins are usually rejected as false positive hits; yet, we reasoned that a proportion of such clones could represent alternative proteins with real affinity for the bait. We found in the literature the partial sequence of five out-of-frame clones from a Y2H experiment performed with the tandem BRCT domain of breast cancer susceptibility protein 1 (BRCA1) [40]. One sequence was 100% identical to an alternative protein from our database whose AltORF is located in the 3′UTR of the mRNA produced from the *MRVI1* gene (Fig. 5A). AltMRVI1^EGFP^ was cloned and transfected into HeLa cells. Similar to BRCA1, AltMRVI1^EGFP^ localized to the nucleus (Fig. 5B). We confirmed the interaction between BRCA1 and AltMRVI1^EGFP^ by co-immunoprecipitation (Fig. 5C). Thus, AltMRVI1 is possibly a novel BRCA1 interacting protein that was already identified by Y2H, but mistakenly rejected as a false positive hit.

**Figure 6:**
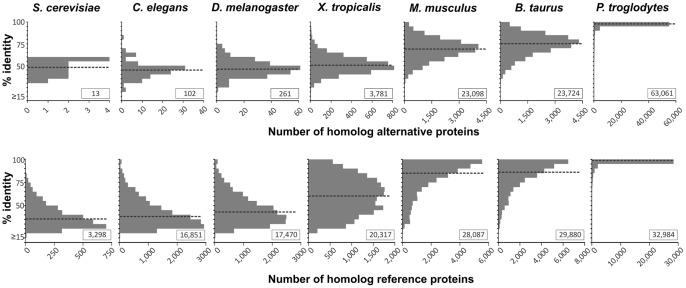
Alternative proteins are conserved from human to yeast. Percent identity of predicted alternative proteins or reference proteins from different eukaryote species analysed with BLASTP (cutoff expectation value of ≤10^−4^) against human corresponding proteins. The number of homologous proteins displaying each percent identity is shown on the y axis. The dotted black bar indicates the median percent identity for each species. Insets indicate the total number of conserved proteins.

To assess the evolutionary conservation of predicted alternative proteins, we generated a database of AltORFs present in mature mRNAs from different eukaryote species (Databases S2−S8), and predicted 5019 distinct alternative proteins in *Saccharomyces cerevisiae*, 35,532 in *Caenorhabditis elegans*, 38,248 in *Drosophila melanogaster*, 52,454 in *Xenopus tropicalis*, 82,305 in *Mus musculus*, 57,492 in *Bos taurus,* and 79,874 in *Pan troglodytes* (Fig. S2; Table S5). The median AltORF size of these predicted proteins ranges from 50 to 57 codons, showing that as observed for humans, the alternative proteome is composed of small proteins compared to the reference proteome (Table S5). Although the homology between reference proteins is greater than that of alternative proteins, we also identified thousands of human alternative proteins conserved with predicted AltORFs in vertebrates, hundreds in invertebrates, and were even surprised to find that 13 alternative proteins are conserved between human and yeast with a median sequence identity of 47.8% (Fig. 6).

## Discussion

The potential of eukaryotic genomes for encoding alternative proteins from non-canonical open reading frames is well known and was recently featured in ribosome profiling studies [34], [41], [42]. Yet, a large scale approach allowing the detection of the corresponding alternative proteins was lacking. In this study, we have generated a database of predicted alternative protein-coding ORFs with a minimum length of 40 codons present in human mRNAs. The data presented here indicate an average of 3.8 AltORFs per human mRNA with a median length of 57 amino acids. Using this database, 1259 human alternative proteins were detected by mass spectrometry in the present study, 3 of which (accession numbers HF548059, HF547970, HF548029) were previously detected [19], [43], [44]. This result strongly supports the hypothesis that the complexity of the proteome has been underestimated and alternative translation initiation already well characterized in viruses cannot be ignored in humans. Importantly, evolutionary conservation of alternative proteins between vertebrates and invertebrate implies that these proteins have significant biological functions.

It is very likely that similar to proteins translated from canonical ORFs, alternative proteins display a wide variety of biological functions. This is suggested by the great diversity of subcellular localizations that we observed in our fluorescence microscopy experiments (Fig. 3, 4C, 5B), and by the fact that a growing number of important functions are attributed to small proteins and peptides [45]−[48]. The polycistronic nature of AltORF encoding mRNAs can potentially lead to intriguing functional interplays between reference and alternative proteins, such as direct interaction between the reference and alternative proteins [11], [49]. There is also evidence that an upstream ORF not only regulates the expression of a downstream RefORF by interfering in *cis* with canonical AUG recognition by scanning ribosomes, but also reduces the translational efficiency of the RefORF in *trans* [45], [50]. AltORFs translation products could also be of particular importance during the thymal selection of T lymphocytes, serving as “cryptic T-cell epitopes”. In some cases, this has been shown to lead to the selection of lymphocytes with antiviral or antitumor activities [51].

Our databases of AltORFs will be useful to identify genes containing multiple protein coding ORFs and to unravel their functions. This is particularly important in experimental settings where gene expression studies (cDNA transfection, knock-down, transgenes, and gene therapy) could result in the expression or down regulation of a reference protein and an unnoticed alternative protein, leading to confounding results [12]. Another striking example is the co-expression of therapeutic transgenes and their associated alternative proteins, which elicit a cytotoxic T lymphocyte response [52]. Thus, transgene sequences should be carefully examined for possible AltORFs to decrease potential adverse immune responses during therapeutic gene transfer.

We also propose that proteins recalcitrant to mass spectrometry identification or proteins with no sequence homology with the conventional proteome identified in large-scale cDNAs screens should be revisited with the AltORFs databases. Additionally, large fundamental and clinical proteomic studies using organs and tissues would likely benefit from the AltORFs database to achieve complete catalogs of proteins in different tissues [53], [54].

The presence of a large fraction of alternative proteins in plasma and serum is particularly interesting as there is a constant need for biomarkers to identify a variety of disorders at an early stage [55]−[57]. The reason why so many alternative proteins are secreted is currently unknown. We did not find any enrichment for classical export signal peptides (not shown), and their secretion mechanism remains to be investigated.

As for any databases, the AltORFs database has some limitations. Although AUG remains the main translation initiation site, recent ribosome profiling studies clearly indicate the use of non-AUG start sites [34]. Yet, we did not take into account non-AUG initiation sites as an accurate prediction method for such functional translation start sites is not yet available [58]. Although there is strong evidence that short peptides are also translated [19], we introduced a cut-off of 40 amino acids to discard from our databases polypeptides shorter than 40 residues, which are less readily detected by conventional mass spectrometry approaches, and to keep the database to a reasonable size. Finally, we used the NCBI reference sequence database (RefSeq) as a source for RNA transcripts. This non-redundant and well-annotated database is fairly conservative, and thus is a quality source for identifying candidate AltORFs, but it would be interesting to compare with other databases (e.g. Gencode) to verify if different mRNA isoforms could also serve as template for the expression of corresponding alternative proteins. Nevertheless, the location of AltORfs is comparable with the distribution obtained in a peptidomic study of small ORFs encoded polypeptides, with the exception of AltORFs located in 5′UTRs [19]. For these AltORFs, the apparent discrepancy probably results from our prediction of AltORFs initiating at AUG sites only [35].

In conclusion, we have provided compelling evidence that alternative proteins significantly contribute to the human proteome by identifying 1259 new proteins and many more will likely be detected in further MS experiments. A comprehensive knowledge of the proteome is of crucial interest to unravel the cellular mechanisms underlying health and disease. We believe that proteomics approaches supported by ribosome profiling will further benefit the establishment of an exhaustive catalog of proteins to fulfill this goal in the future.

## Supporting Information

Figure S1 **Hematoxylin, eosin and saffron-stained sections of normal and cancerous tissues in two patients.** Patient 1, sections of normal and serous ovarian tissues and normal and serous cancerous fallopian tissue. Patient 2, sections of normal, borderline and endometrioid ovarian tissues and normal and hyperplasic endometrial tissues. Annotations of the tissues were performed by a pathologist (Dr. O. Kerdraon, Centre Oscar Lambret, Lille, France).
(TIF)

Figure S2 **AltORFs distribution among eukaryote species.** Distribution in % across the different mRNA regions. The number of distinct proteins predicted for each species is displayed in the insert.
(TIF)

Table S1 **Alternative and reference proteins list in diverse cell lines.** For a given alternative protein, N-terminal N-acetylated peptides and sequence coverage (%) are indicated in additional columns. When co-expression of the reference and alternative proteins is observed for a particular gene, the lane is highlighted in gray. The sequence covered by all detected peptides is underlined in the alternative protein amino acid sequence column. For the HeLa cell line whole-protein analysis, a total of 129 alternative proteins identified by 280 peptides, and 5,558 reference proteins identified by 68,035 peptides were detected. For the HeLa cell line analysis of proteins between the 4.6 and 10 kDa marker of a 1-D SDS-PAGE, a total of 14 alternative proteins identified by 18 peptides, and 44 reference proteins identified by 109 peptides were detected. We excluded from the identified proteins those with expected molecular weights above 10 kDa since fragments below 10 kDa likely represent breakdown products. For the colon cell lines, a total of 45 alternative proteins identified by 63 peptides, and 3,512 reference proteins identified by 39,285 peptides were detected.
(XLSX)

Table S2 **Alternative and reference proteins list in human tissues.** For a given alternative protein, N-terminal N-acetylated peptides and sequence coverage (%) are indicated in additional columns. The sequence covered by all detected peptides is underlined in the alternative protein amino acid sequence column. For the colon tissue, a total of 13 alternative proteins identified by 17 peptides, and 1,985 reference proteins identified by 16,068 peptides were detected. For the lung tissue, a total of 40 alternative proteins and 2,373 reference proteins were detected. For the normal endometrium, endometrioid ovary, serous ovary, normal ovary, and serous fallopian tube, a total of 19 alternative proteins and 2,748 reference proteins were detected.
(XLSX)

Table S3 **Alternative and reference proteins list in human fluid.** For a given alternative protein, N-terminal N-acetylated peptides and sequence coverage (%) are indicated in additional columns. The sequence covered by all detected peptides is underlined in the alternative protein amino acid sequence column. In cerebrospinal fluid, a total of 16 alternative proteins and 266 reference proteins were detected. In urine, a total of 47 alternative proteins and 754 reference proteins were detected. In plasma, a total of 90 alternative proteins and 70 reference proteins were detected. In serum. a total of 928 alternative proteins and 754 reference proteins were detected.
(XLSX)

Table S4 **Combined alternative proteins list.** All alternative proteins identified across all analysed samples are shown. For a given alternative protein, if N-terminal N-acetylated peptides were detected, they are indicated in an additional column and the entire lane is highlighted in gray. The sequence covered by all detected peptides is underlined in the alternative protein amino acid sequence column.
(XLSX)

Table S5 **Summary of AltORFs and alternative proteins characteristics in different eukaryote species.**
(DOC)

Database S1 **A database of alternative ORFs in** ***Homo sapiens.*** For each AltORF, the gene name and accession number of the mRNA in which it is encoded are provided. Other information can also be found for both the reference ORF and the alternative ORF, including the reading frame and the coordinates of the start and stop codon (with respect to the first nucleotide of the mRNA). The predicted amino acid sequence of the alternative protein is also indicated.
(XLSX)

Database S2 **A database of alternative ORFs in** ***Pan troglodytes*** **.** For each AltORF, the gene name and accession number of the mRNA in which it is encoded is provided in the first tab. Other information can also be found for both the reference ORF and the alternative ORF, including the reading frame and the coordinates of the start and stop codon (with respect to the first nucleotide of the mRNA). The predicted amino acid sequence of the alternative protein is also indicated. The second tab displays the results of alternative protein conservation analysis against human. The gene name and accession number for chimpanzee and human homolog proteins are provided, as well as standard BLASTP output, and similarity percentage.
(XLSX)

Database S3 **A database of alternative ORFs in** ***Mus musculus*** **.** For each AltORF, the gene name and accession number of the mRNA in which it is encoded is provided in the first tab. Other information can also be found for both the reference ORF and the alternative ORF, including the reading frame and the coordinates of the start and stop codon (with respect to the first nucleotide of the mRNA). The predicted amino acid sequence of the alternative protein is also indicated. The second tab displays the results of alternative protein conservation analysis against human. The gene name and accession number for mouse and human homolog proteins are provided, as well as standard BLASTP output, and similarity percentage.
(XLSX)

Database S4 **A database of alternative ORFs in** ***Bos taurus*** **.** For each AltORF, the gene name and accession number of the mRNA in which it is encoded is provided in the first tab. Other information can also be found for both the reference ORF and the alternative ORF, including the reading frame and the coordinates of the start and stop codon (with respect to the first nucleotide of the mRNA). The predicted amino acid sequence of the alternative protein is also indicated. The second tab displays the results of alternative protein conservation analysis against human. The gene name and accession number for cow and human homolog proteins are provided, as well as standard BLASTP output, and similarity percentage.
(XLSX)

Database S5 **A database of alternative ORFs in** ***Xenopus tropicalis*** **.** For each AltORF, the gene name and accession number of the mRNA in which it is encoded is provided in the first tab. Other information can also be found for both the reference ORF and the alternative ORF, including the reading frame and the coordinates of the start and stop codon (with respect to the first nucleotide of the mRNA). The predicted amino acid sequence of the alternative protein is also indicated. The second tab displays the results of alternative protein conservation analysis against human. The gene name and accession number for frog and human homolog proteins are provided, as well as standard BLASTP output, and similarity percentage.
(XLSX)

Database S6 **A database of alternative ORFs in** ***Drosophila melanogaster*** **.** For each AltORF, the gene name and accession number of the mRNA in which it is encoded is provided in the first tab. Other information can also be found for both the reference ORF and the alternative ORF, including the reading frame and the coordinates of the start and stop codon (with respect to the first nucleotide of the mRNA). The predicted amino acid sequence of the alternative protein is also indicated. The second tab displays the results of alternative protein conservation analysis against human. The gene name and accession number for fly and human homolog proteins are provided, as well as standard BLASTP output, and similarity percentage.
(XLSX)

Database S7 **A database of alternative ORFs in** ***Caenorhabditis elegans*** **.** For each AltORF, the gene name and accession number of the mRNA in which it is encoded is provided in the first tab. Other information can also be found for both the reference ORF and the alternative ORF, including the reading frame and the coordinates of the start and stop codon (with respect to the first nucleotide of the mRNA). The predicted amino acid sequence of the alternative protein is also indicated. The second tab displays the results of alternative protein conservation analysis against human. The gene name and accession number for nematode and human homolog proteins are provided, as well as standard BLASTP output, and similarity percentage.
(XLSX)

Database S8 **A database of alternative ORFs in** ***Saccharomyces cerevisiae*** **.** For each AltORF, the gene name and accession number of the mRNA in which it is encoded is provided in the first tab. Other information can also be found for both the reference ORF and the alternative ORF, including the reading frame and the coordinates of the start and stop codon (with respect to the first nucleotide of the mRNA). The predicted amino acid sequence of the alternative protein is also indicated. The second tab displays the results of alternative protein conservation analysis against human. The gene name and accession number for yeast and human homolog proteins are provided, as well as standard BLASTP output, and similarity percentage.
(XLSX)
