# Supplementary figures and images for: Direct Detection of Alternative Open Reading Frames Translation Products in Human Significantly Expands the Proteome
Source: PLoS One. 2013 Aug 12;8(8):e70698. doi: 10.1371/journal.pone.0070698 (PMC3741303; doi:10.1371/journal.pone.0070698)

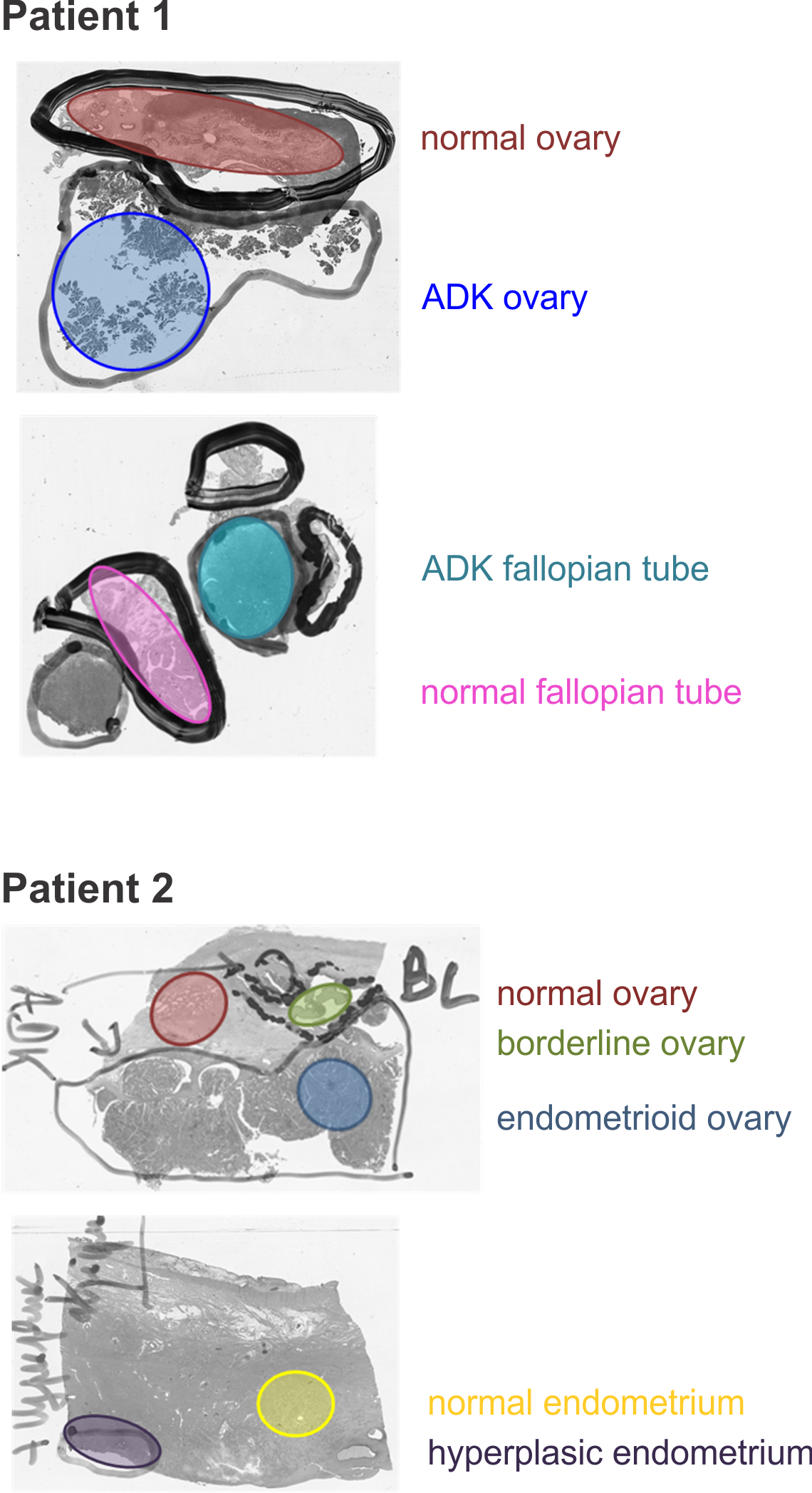

Supplement: Figure S1 — Hematoxylin, eosin and saffron-stained sections of normal and cancerous tissues in two patients. Patient 1, sections of normal and serous ovarian tissues and normal and serous cancerous fallopian tissue. Patient 2, sections of normal, borderline and endometrioid ovarian tissues and normal and hyperplasic endometrial tissues. Annotations of the tissues were performed by a pathologist (Dr. O. Kerdraon, Centre Oscar Lambret, Lille, France). (TIF) [file pone.0070698.s001.tif]

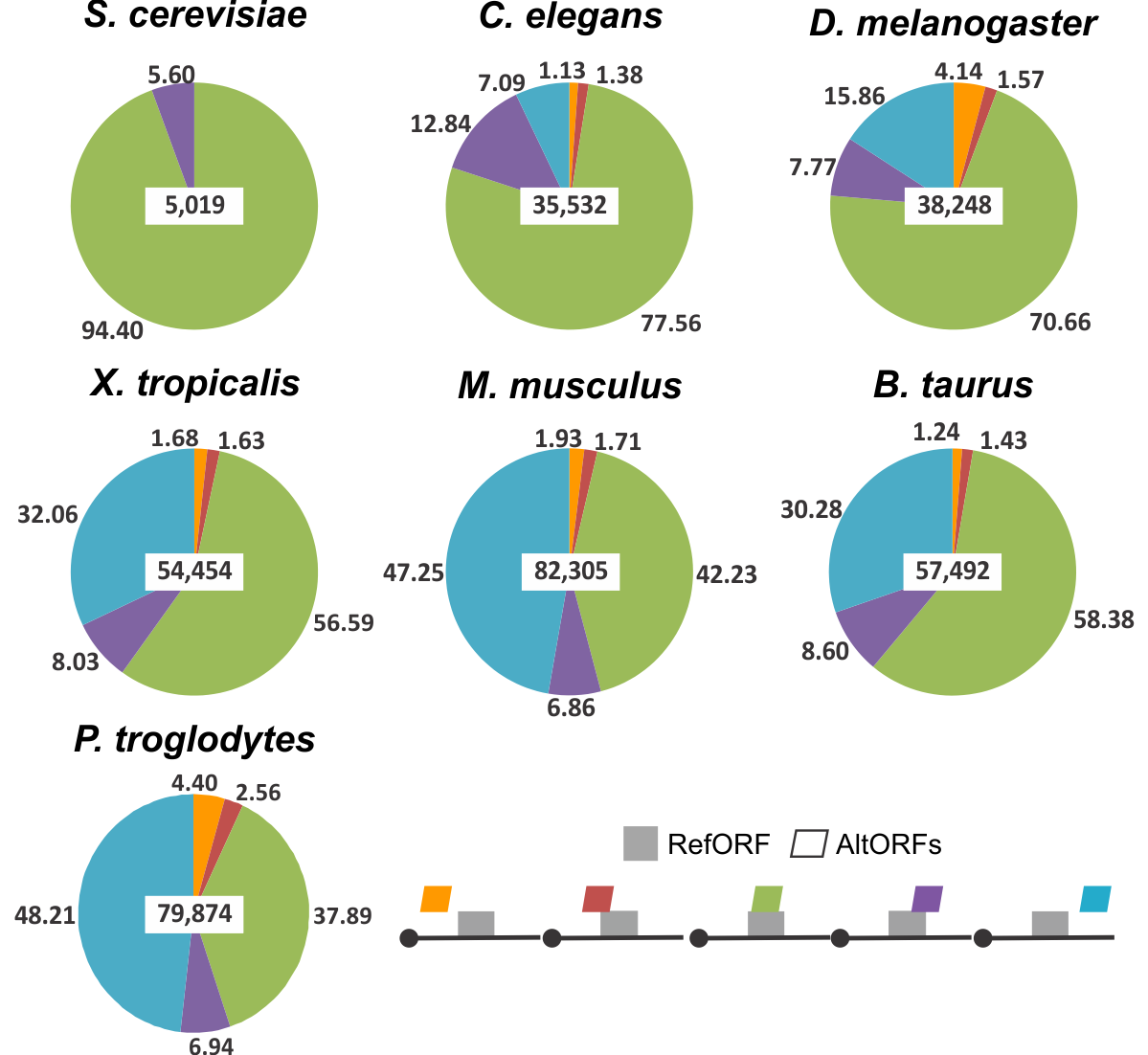

Supplement: Figure S2 — AltORFs distribution among eukaryote species. Distribution in % across the different mRNA regions. The number of distinct proteins predicted for each species is displayed in the insert. (TIF) [file pone.0070698.s002.tif]
