# Supplementary material for: Direct Detection of Alternative Open Reading Frames Translation Products in Human Significantly Expands the Proteome
Source: PLoS One. 2013 Aug 12;8(8):e70698. doi: 10.1371/journal.pone.0070698 (PMC3741303; doi:10.1371/journal.pone.0070698)
Supplement: Table S5 — Summary of AltORFs and alternative proteins characteristics in different eukaryote species. (DOC) [file pone.0070698.s007.doc]

**Table S5.** **Summary of AltORFs and alternative proteins characteristics in different eukaryote species**

| **Species** | **mRNAs^a^** | **% of mRNAs**  **with AltORF(s)** | **#AltORFs/**  **mRNA^b^** | **Total AltORFs** | **Distinct alternative proteins^c^** | **AltORFs size^b^**  **(# codons)** | **RefORFs size^b^**  **(# codons)** |
| --- | --- | --- | --- | --- | --- | --- | --- |
| *S. cerevisiae* | 6,692 | 47.37 | 0.79 | 5,288 | 5,019 | 50 | 359 |
| *C. elegans* | 32,693 | 72.62 | 1.89 | 61,922 | 35,532 | 56 | 346 |
| *D. melanogaster* | 24,019 | 83.06 | 3.18 | 76,401 | 38,248 | 57 | 455 |
| *X. tropicalis* | 22,472 | 78.53 | 2.43 | 54,661 | 52,454 | 53 | 386 |
| *B. taurus* | 32,229 | 62.74 | 2.39 | 76,925 | 57,492 | 57 | 360 |
| *M. musculus* | 28,853 | 85.43 | 3.80 | 109,774 | 82,305 | 56 | 397 |
| *P. troglodytes* | 33,850 | 85.23 | 3.75 | 126,875 | 79,874 | 55 | 384 |

**^a^**Number of transcripts used to generate the database

**^b^**Median value

^c^Since an AltORF may be present in several transcripts, the total number of AltORFs in the transcriptome exceeds the number of distinct alternative proteins
